# Clinicians’ assumptions about Sami culture and experience providing mental health services to Indigenous patients in Norway

**DOI:** 10.1177/1363461520903123

**Published:** 2020-02-06

**Authors:** Inger Dagsvold, Snefrid Møllersen, Bodil H Blix

**Affiliations:** 1 UiT The Arctic University of Norway; 2 Finnmark Hospital Trust, Norway

**Keywords:** Cultural adaption, indigenous, mental health service, Norway, qualitative research, Sami

## Abstract

This qualitative study explores Sami and non-Sami clinicians’ assumptions about Sami culture and their experiences in providing mental health services to Sami patients. The aim is to better understand and improve the ways in which culture is incorporated into mental health services in practice. Semi-structured interviews were conducted with 20 clinicians in mental health outpatient clinics in the northern Sami area in Troms and Finnmark County in Norway. The findings show that clinicians’ conceptualizations of culture influence how they take cultural considerations about their Sami patients into account. To better integrate culture into clinical practice, the cultures of both patient and clinician, as well as of mental health care itself, need to be assessed. Finally, the findings indicate a lack of professional team discussions about the role of Sami culture in clinical practice.

## Introduction

Culture matters in mental health care because it shapes the experience and expression of mental health problems, as well as health-related beliefs, help-seeking behaviors and ideas about treatment (Helman, 2007; Kirmayer, 2012; Kleinman & Benson, 2006). Cultural differences are often used as an explanation for why minority populations and indigenous people are less satisfied with health services than majority populations (Alizadeh & Chavan, 2016; King, Smith, & Gracey, 2009). In Norway, studies indicate that the indigenous Sami population experience more communication problems and are less satisfied with mental health services than the majority population (Dyregrov, Berntsen, & Silviken, 2014; Møllersen, 2007; Sørlie & Nergård, 2005). The Sami people in Norway have a statutory right^1^ to receive equitable health services, and “Sami cultural competence” among health professionals is described in government reports as the means to achieve this aim (Ministry of Health and Care Services, 2009; Ministry of Health and Social Affairs, 1995; Ministry of Labour and Social Affairs, 2008). The concept of cultural competence is often seen as a tool to bridge cultural differences and enable the provision of “culturally adapted” health services to patients with “diverse values, beliefs and behaviors [and] meet patients’ […] cultural […] needs” (Betancourt, Green, & Carrillo, 2002, p. v). However, limited research is available on the provision of culturally adapted mental health services to Sami patients. The imposed responsibility to provide culturally adapted health care to the Sami is non-specified and applies for all parts of the health services. To our knowledge, there is no research showing that any specific diagnosis or treatment directions require Sami cultural facilitation of mental health care or are more suitable for it than others. Therefore, in this study, we explore clinicians’ assumptions about Sami culture and their experiences with providing “culturally adapted” mental health services to Sami patients in a wide sense.

The concept of culture is complex and multiple definitions exist (Browne & Varcoe, 2009; Kroeber & Kluckhohn, 1952; Sobo, 2009), several of which are variations on Tylor’s definition from 1871: “Culture, or civilization […] is that complex whole which includes knowledge, belief, art, morals, law, custom, and any other capabilities and habits acquired by man as a member of society” (Tylor, 2016 [1871], p. 10).

Tylor’s definition is often understood and used in an essentializing way that represents culture as a static set of beliefs and practices of groups of people. The theorization of culture, however, has changed over time, and culture has come to be viewed as dynamic, complex, ambiguous, related to world-view and “meaning-making,” and closely related to social interaction, historical and political factors, and power structures (Barth, 1969, 1994; Helman, 2007). Culture is also described as patterns of thought, communication, and behavior (Schackt, 2009). And while common cultural patterns may be influential, they do not determine individuals’ mindsets and modes of living, nor are they out of reach of conscious reflection. Considerable within-group cultural differences and individual variations exist. Within nursing and health care, however, the tendency is to view culture in an essentializing way, assuming that patients of a certain ethnic group “possess a particular set of [cultural] attributes or traits, about which clinicians can be trained to develop cultural competence” (Vandenberg 2010, p. 241; Blix, 2014; Schackt, 2009).

Many models and programs for cultural competence have been developed and the role of culture and cultural adaptation in the provision of health care to indigenous patients is increasingly discussed (Good & Hannah, 2015; Kirmayer, 2012; Kleinman & Benson, 2006; Sobo, 2009). The cultural competence concept has been criticized for its essentializing view of culture, presupposing that individuals of a cultural or ethnic group think, feel, or act in certain ways, thereby failing to consider the individuals’ life histories or social contexts. An essentialized view of culture risks ignoring complexity and other significant factors, such as gender, education, class, economy, and geographical location (Kirmayer, 2012; Kleinman & Benson, 2006, p. 1673). Cultural competence is also criticized for “othering,” i.e., focusing solely on the culturally different “others” and ignoring the significance of the cultures of health services and clinicians. Moreover, evidence of the benefits and efficiency of culturally competent care is lacking (Alizadeh & Chavan, 2016; Browne & Varcoe, 2009; Kirmayer, 2012; Kleinman & Benson, 2006). Additionally, descriptions of how to operationalize cultural competence in clinical practice without reducing holistic care to “technical skills for which clinicians can be trained to develop expertise in how to treat a patient of a given ethnic background” (Kleinman & Benson, 2006, p. 1673) are limited. Our aim here is not to assess different models of cultural competence but rather to reflect on the process through which cultural considerations become integrated into health care. This can inform efforts to improve the integration of culture into mental health care.

## Methods

We conducted qualitative interviews with clinicians who are obliged to integrate cultural considerations into their practice. The study included clinicians providing therapy to Sami patients in outpatient mental health clinics. We requested permission to interview clinicians from seven mental health clinics serving patients in the Sami Language Administrative District^2^ in Northern Norway; three clinics consented, all of which are located in the northern Sami area. We organized informational sessions, distributed written information about the study, and extended an invitation to participate to 60 clinicians during 2012–2013. The only inclusion criterion was experience providing mental health care to Sami patients. Clinicians interested in participating submitted the consent form to the first author, who made appointments for interviews. Twenty clinicians agreed to participate, and all were included in the study.

The clinicians included in the study were nine men and 11 women, aged between mid-20 s and late 60 s. Eleven participants self-identified as Sami and nine as non-Sami. Five participants, both Sami and non-Sami, spoke Northern Sami language fluently and could provide treatment in Sami, whereas 15 were unable to provide treatment in Sami. The participants had lived in a Sami area for between one year and all of their lives. In terms of their professional backgrounds, 10 were qualified nurses, social workers, physiotherapists, or occupational therapists, and 10 were psychologists, clinical psychologists, or psychiatrists. Three of the participants had attended short courses in cultural studies. None of them had formal cultural competence training. Their work experience in mental health care ranged from two to almost 40 years. The study does not include information about the clinicians’ patients or clinical approaches.

### Data collection

The interviews were conducted by the first author and took place at locations chosen by participants (usually their workplace), lasting from 50 to 140 minutes. The semi-structured thematic interview guide included items concerning assumptions about Sami culture and the integration of Sami culture into mental health care. Questions concerning the use of Sami vs. Norwegian language in therapy were discussed in the interviews and the results are presented in other publications resulting from this study (Dagsvold, Møllersen, & Stordahl, 2015, 2016). The interview questions were open-ended and the order flexible. Participants were encouraged to talk freely and to draw on their experiences. All interviews were conducted in Norwegian because the interviewer did not speak Sami fluently. The interviewer offered to use an interpreter but all participants wished rather to conduct the interview in Norwegian. The interviews were audiotaped and transcribed verbatim.

### Analysis

The transcribed texts were analyzed thematically using an inductive approach and systematic text reduction (Malterud, 2001, 2011, 2012). All transcripts were read several times to obtain a general impression and preliminary themes were identified. The texts were then systematically examined and units of meaning were identified. The meaning units for each participant were condensed and coded. The codes were systematized and categorized, and related codes were sorted into themes and subthemes. Finally, short text summaries based on our interpretations of these themes formed the basis of the results. The first author read all the interview transcripts and selected half of the interviews for the third author to read. Furthermore, the first author created code groups and themes, which were introduced to the co-authors along with selected quotations. The code groups and themes were then modified and further developed by all authors.

### Ethical considerations

The research protocol was approved by the Regional Committees for Medical and Health Research Ethics (REC)^3^ and was conducted in accordance with the Helsinki Declaration of 1975, revised in 2008. To safeguard the participants’ anonymity, personal details are not included in the presentation of the findings.

## Results

The analysis identified two major themes: the clinicians’ assumptions and descriptions of Sami culture more generally, and the impact of Sami culture on the therapeutic encounter. The analysis did not show overall differences in assumptions of culture or clinical experiences based on the participants’ ethnicity, gender, or education.

### Theme I: Clinicians’ assumptions and descriptions of Sami culture

When referring to Sami culture, the participants discussed three subthemes: 1) cultural traits of the Sami way of life, 2) the Sami way to communicate, and 3) Sami attitudes toward mental illness.

#### Cultural traits of the Sami way of life

When referring to Sami culture, both Sami and non-Sami participants stated that Sami live close to nature in geographically remote areas, that their traditional occupations are reindeer herding and fishing, and that their family structure is the extended family. They also mentioned Sami people’s preference to keep problems within the family. Moreover, the participants typically mentioned Sami arts, handicrafts (*duodji*), music (*joik*), and traditional clothing (*gákti*) when referring to Sami culture. They also mentioned key historical and political aspects as relevant to Sami society, particularly forced assimilation (the “Norwegianization process”) including stigmatized identity and language loss. A non-Sami participant stressed the impact of having knowledge about the history and local society to understand the patients in a better way:

We need to know more about the history, and how our patients live, this applies not only to the inland, but to the coast as well … the Sea Sami … those who have lost the language, who feel like Sami, but do not speak Sami … It’s important to know a little more about that … how people feel about that.

#### The Sami way to communicate

Several participants stated that the Sami communicate in a “Sami way.” The Sami way to communicate included communication style, absence of verbal communication about certain topics, and an ability to communicate with deceased people. According to the participants, the Sami communication style is indirect or in a slow manner, and they keep long-lasting eye contact and use metaphors, body language, and silence. Moreover, the Sami way was referred to as not talking about emotions and mental illness and being able to communicate with the deceased. The participants stated that communicating with the deceased, especially their relatives, is a common phenomenon considered normal in a Sami cultural context. One Sami participant stated: “Talking to a deceased grandmother is a Sami tradition, it is a coping strategy and the person feels protected.” Another Sami participant referred to this type of communication as an “inner conversation with a trusted person, which may increase self-reflection and self-understanding.”

#### Sami attitudes toward mental illness

Several participants, both Sami and non-Sami, stated that the Sami have “old-fashioned attitudes toward mental problems and receiving treatment,” and more specifically that the Sami consider mental illness to be a weakness and a shameful thing. Some claimed that the Sami solve their problems within the family; however, most of the participants stated that the Sami prefer to manage on their own (*ieš birget*), and avoid seeking help from either family members or mental health services. Consequently, in their opinion, the Sami “do not talk about emotions and mental illness,” and they “hide their problems [and do not ask for help] because one is not supposed to show weakness.”

Most participants, both Sami and non-Sami, referred to Sami culture by pointing out distinct cultural characteristics. The rather stereotypical descriptions of Sami culture point to a historical and narrow perception of the Sami. As such, the Sami appear as different from an implicit and invisible “norm of normality,” or standard—in this case, the Norwegian majority population. However, the basis of comparison remained unspoken. An implicit comparison with an invisible norm of normality positions the Sami as something deviant from the “standard”; that is, the others, who behave in a certain “Sami way.”

### Theme II: The impact of Sami culture on therapy

Although most participants described what they assumed to be Sami culture, only a few elaborated on how their clinical encounters were influenced by it. We identified three ways in which the few clinicians reflected on the impact of Sami culture on their clinical work: 1) clinical experiences nuance assumptions of a Sami communication style; 2) in correspondence with: Assessing Sami patients' experience: Cultural phenomenon or symptom of disease?; and 3) cultural considerations are not part of professional discussions in the clinic.

#### Clinical experiences nuance assumption of a Sami communication style

Although several Sami and non-Sami participants described a distinct Sami way to communicate when speaking in general terms, their clinical experience with Sami patients’ communication style was more nuanced. When talking about their clinical experiences, they stated that Sami individuals did seek mental health services, and they did talk about problems and emotions in therapy. However, some pointed out that Sami cultural norms may influence what Sami patients talk about in different contexts and how mental health issues are worded or expressed. Some participants stated that the Sami norm of “not talking” might restrain communication about mental health problems in public, in Sami communities, and in some families. Also, some participants noted that Sami patients themselves occasionally initiated the consultations by stating that “the Sami do not talk about emotions and mental health issues” but nonetheless continued to talk about such issues in therapy. One non-Sami participant elaborated on this statement:

Sami patients are as communicative as Norwegian patients once they’ve come in here. The ones who come here have acknowledged that they have a problem they need help with. So my main impression is that there’s not such a big difference, we have good talks.

This non-Sami participant framed Sami patients’ willingness to speak during therapy as adapting to the context and accepting the “rules of the game” of therapy, where communication about sensitive matters is a significant aspect:

I think something happens when they get into our offices, it’s our chairs and tables, you know. And we sit right opposite each other, kind of business-like. They change, they take on the role of a patient, and of course they too have an idea about what the session is for.

However, some participants’ experiences correspond with general statements and assumptions about Sami culture. One non-Sami participant stated that Sami patients may well speak in metaphors or indirectly “when they find it difficult to talk about sensitive matters.” Another Sami participant noted that Sami patients may frame mental health problems differently from the “psychological way” by saying that “something’s getting worn out, more like a practical problem than a psychological problem.” This participant defined the “psychological way” just as cultural as the Sami way: “It seems to me there are really two different cultural ways to talk about how you feel.”

One non-Sami participant remarked that the Sami communication style influences the clinical interview and the recording of a person’s medical histories:

There aren’t many Sami who are very spontaneous, they don’t tell you things spontaneously, so you have to drag their medical history out of them … things move a lot more slowly than I’m used to. They generally talk more slowly, … and sometimes they’ll keep eye contact with you for a long time before saying anything […].

This participant maintained that the “slow communication” was a “Sami way” of communicating, without reflecting on other aspects such as language problems, lack of habit to talk about certain issues, individual personality, or the relational aspect of the communication. Consequently, the possibility of the patient’s “slow” communication being a response to, for example, the therapists’ cultural conduct rather than a Sami cultural trait was not discussed.

#### Assessing Sami patients’ experience: Cultural phenomenon or symptom of disease?

Several participants stated that they had heard that the Sami “talk to deceased people.” However, only a few participants reported that they had met Sami patients in therapy who stated that they were communicating with the dead. These participants emphasized the necessity of exploring such experiences to make an adequate clinical assessment of whether this behavior is a symptom of illness, affecting the patients’ “functioning,” or alternatively, a common phenomenon in the patients’ cultural context.

A Sami participant suggested “entering into the patient's stories” and including the deceased in clinical communication, for example by asking the patient: “What would Grandmother say about this?” Another Sami participant described communication with the dead as an integral theme of her initial interviews in therapy:

To me it’s natural to ask about such things [talking to dead people] when you take a patient’s history, at least if you want to find out about the family. Then you have to include things like that and if it’s a deceased grandmother who’s important, however dead she is, well then that’s important information.

A non-Sami participant compared talking with the deceased with obsessive thoughts that potentially could hinder recovery, and suggested that the therapy should aim to remove such nonfunctional strategies:

The feeling that “Grandma’s looking after me,” well, I’d call that a security strategy that becomes an obstacle for daring to do things on your own and will prevent you from getting well. In compulsive thinking, it’s often about like if I do something magical, things will be ok. So if you think Grandmother’s there and that’s why things turned out ok, you won’t get rid of your anxiety. Because if your grandmother suddenly isn’t there one day, you’re just as afraid. So, whether this security strategy is inside your head or you’re taking Valium, it’s the same phenomenon.

Other participants assessed their Sami patients’ communication with the deceased as a symptom of mental illness if it tormented them or affected their functioning negatively. Some participants addressed the impact of clinicians’ perceptions of illness and their power to define “normality.” For example, one non-Sami participant stated:

It’s very much about the view of the work we do in mental health care. Who is the person we meet, are we meeting normality ... we have different explanatory models, we have a disease model where everything is pathology, and then talking to your dead father is pathological, it’s a delusion. […] So health care and psychiatry, … it gets so one-sided in terms of disease and diagnosis and you get caught up in that limited life that is your diagnosis. It seems like it’s a bit about roles, about power, I mean the power of definition in relation to normality.

The risks associated with automatically jumping to cultural explanations were mentioned by a Sami participant, who emphasized the necessity of carefully exploring the patient’s experience:

As therapists, we must be aware of what something is, you shouldn’t think straightaway, “Oh yes, well, this is Sami culture, so it’s okay,” or say, “No, we mustn’t talk about that.” We need to explore it, talk about it, and ask how the patient experiences it, what does it mean to the patient, is it a problem, does it affect your functioning, we have to make an assessment and look at the whole situation ... and decide if the patient is actually becoming psychotic. You can’t rule out anything. We have a responsibility to assess and treat our patients, so you can’t just say something’s a cultural phenomenon without examining it, so it’s a balancing act. It’s not one or the other, it’s a bit of everything.

The therapists’ clinical experiences illustrate the complexity in assessing patients’ experiences and expressions as normal in the patients’ cultural context or as a symptom within mental health treatment. The participants did not mention specific diagnoses or treatment programs as particularly relevant for cultural adaption.

#### Cultural considerations are not part of professional discussions in the clinic

According to most participants, cultural considerations are not part of professional discussions at their workplaces. One Sami participant stated: “we don’t have [team] discussions about individual patients where we include cultural aspects, [asking] what’s important to consider, what is this, what can we do, what about doing things differently …” Another Sami participant described attempts at integrating cultural issues in team discussions as unsuccessful: “We [tried but] got off track, it just ended up as a professional [mental health] issue, but I think this culture is also a professional issue.” Information about patients’ culture was seen as a necessity to ask informed questions in therapy and to explore individual patients’ experiences and way of life. One non-Sami participant said:

We have to get hold of what the patients’ experience is, ask patients to tell us about their everyday lives, because people’s own stories are more important than general ideas about culture. [However,] you have to know a lot about the culture to listen closely to the conversations to find leads that are important to follow …

Another non-Sami participant expressed concerns that an exaggerated focus on Sami culture in team discussions and patient assessments could divert attention from the individual patients’ needs and preferences. This non-Sami participant stated that: “When the focus is on culture, you don’t get the chance to gain access to the person, the individual patient. That would be a strange approach to our work that I’m not comfortable with.” Overall, the findings show that few participants had experiences in which they felt that their clinical assessment of, and communication with, Sami patients was influenced by Sami culture. Those who did refer to such experiences reported that Sami patients do not always act in accordance with assumptions about Sami culture. Their general descriptions of Sami culture did not correspond with the lifestyle and behavior of individual Sami patients. Moreover, the participants reported little or no training in how to incorporate aspects of Sami culture into mental health care and a lack of discussion within their teams about the role of Sami culture in their practice.

## Discussion

This study aimed to explore clinicians’ assumptions about Sami culture in general and how in their clinical practice cultural considerations are taken into account to provide culturally adapted mental health services. We will discuss the results in relation to culture as essential traits characterizing a group and as an individual dynamic and contextual process, and reflect on possible consequences for clinical practice.

The way culture is understood and conceptualized influences the approach to its integration into health care (Guarnaccia & Rodriguez, 1996). In our study, we found that the clinicians referred to Sami culture predominantly in terms of particular “cultural traits” typically considered “different” and implicitly compared with an unspoken norm of the “ordinary.” Several of the Sami cultural traits described in our study concur with the representations in Norwegian media and public discourse. Here, the Sami are often presented as “the exotic others”; a “natural people,” who live close to nature as nomadic reindeer herders or as sea Sami fishers in rural Sami areas. Such descriptions of Sami culture reflect an essentialized view of culture as static and narrow in scope. Through these descriptions, the Sami are perceived to hold certain “authentic” qualities, different from and in contradiction to modern, “civilized” peoples and societies (Gaski, 2008; Kvidal-Røvik & Olsen, 2016; Mathisen, 2001). Essentialized descriptions do not reflect the fact that contemporary Sami societies are as complex and diverse as other societies. The Sami population and their needs and preferences are heterogeneous. Indeed, less than 10% of the Sami are engaged in reindeer husbandry, many Sami live outside the so-called Sami core areas, and most Sami do not speak a Sami language or possess visible cultural markers (Gaski, 2008; Sørlie & Broderstad, 2011; Sørlie, Hansen, & Friborg, 2018). Moreover, clinicians’ descriptions of Sami culture in our study concur with those in the health-related literature, which reports particular “Sami attitudes” toward mental health problems. This literature holds that Sami people consider mental illness to be a shameful matter and thus do not talk about mental health problems or emotions, prefering to manage on their own (*ieš birget*), and that they can communicate with the deceased (Bongo, 2012; Ministry of Health and Care Services, 2015; Ministry of Health and Social Affairs, 1995; Nymo, 2011; Sexton & Sørlie, 2009).

One problem with an essentialized approach to culture in health care is that health professionals are taught to look for specific cultural traits when trying to identify patients who might need “culturally adapted” care (Browne, 2005). Consequently, clinicians may refrain from reflecting on patients’ cultural backgrounds and fail to identify cultural aspects of clinical relevance unless they fit the stereotypical characteristics of Sami culture. In a previous study, clinicians identified patients’ Sami language competence only if they observed what they considered typical Sami characteristics, such as speaking Norwegian with a Sami accent, “looking like a Sami,” or having a typical Sami name or place of residence; they failed to identify Sami-speaking patients from the coast, who therefore did not receive language appropriate mental health care (Dagsvold et al., 2016).

Another problem with cultural essentialism is that it implies culturalism; for example, anticipation that Sami patients will act in accordance with their culture in therapy—in our case, not talking about mental illness. Essentialized descriptions and a static view on culture ignore individual innovation and the dynamics of culture. A dynamic approach to culture promotes individual choices and holds that cultural norms and values are not static. For example, in contrast to their assumptions about Sami culture, some clinicians in our study reported no clinical differences and stated that Sami patients do in fact talk about their problems. Some reported that Sami patients occasionally state “the Sami do not talk about …” before starting to talk about it themselves. This example illustrates that cultural ideas and practices may have different meanings for individuals in different situations and must be understood in the context in which they appear (Sobo, 2009). In our case, the norms of “*ieš birget*” and “not talking about” do not simply transfer to another context; transfer of cultural norms from one context to another must therefore be done with caution. “*Ieš birget”* and not talking about personal problems might be productive within the context of primary industries such as reindeer herding and among fishers, who work under harsh conditions and are forced to fend for themselves out on the tundra or at sea. However, such cultural norms may not be adequate in the context of mental health care. Assuming that members of an ethnic group share cultural meanings may lead to dangerous stereotyping (Kleinman & Benson, 2006) and lack of proper health care. Blix & Hamran (2017) illustrated that clinicians’ cultural assumptions led them to attribute the reluctance of Sami service users to seek and accept help to their culture, and therefore did not intervene toward patients’ health needs. Assumptions that Sami patients act in accordance with Sami culture ignore other possible explanations as to why patients seemingly prefer to “*ieš birget*” or appear less talkative. This behavior may be explained by a lack of language choice in therapy (Dagsvold et al., 2016), unfamiliarity with talking about certain issues, reluctance to share personal matters with clinicians in small communities with close and multiplex relations (Dagsvold et al., 2015; Dyregrov et al., 2014), lack of trust, or simply individual preferences. Additionally, cultural (or personal) differences in communication style and norms between the patient and the clinician may lead to misunderstandings. Some clinicians in our study stated that the Sami “do not talk about mental problems” or referred to the Sami way to communicate as indirect, slow, metaphorical, nonverbal, or silent. According to Vandenberg (2010), “continued emphasis on ‘difference’ [in ‘the other’] can distract from the complexities of relationship building” (p. 243). Assertions about the “Sami way,” such as the clinician in our study who stated that that it was necessary to “drag the words out of the Sami” because the Sami speak in a “Sami way,” disregard the relational aspect of communication and interaction and ignore the impact of the clinicians’ own cultural way of communicating on the therapy. Dyregrov et al. (2014) present the possibility that Sami clinicians may have internalized the norms of not talking about certain issues, and may be unaccustomed to or uncomfortable with talking about what they themselves consider sensitive issues in therapy with Sami patients. The authors describe that a bereaved Sami criticized a Sami therapist for not talking about a sensitive issue—sudden death in the family—therefore not providing proper health care to the bereaved. The authors suggest that Sami clinicians can be less talkative if they have internalized the Sami norms of not talking about culturally sensitive issues. Moreover, other aspects may be mistaken for culture, influencing the therapy. When working in small and multiplex societies, clinicians may have a non-professional relationship to the service user, or be personally affected by the death, therefore not being able to talk about the issue.

Assessing the impact of culture on individuals’ behavior is a complex matter. Occasionally individuals’ behavior complies with stereotypical assumptions of culture; in other cases they do not. In Dyregrov et al. (2014), the Sami service user wanted to talk about the sudden death, but experienced that the Sami clinician avoided or ignored this wish. In a previous study, a Sami patient receiving mental health care stated that she had to adapt her way of communicating, that is, be more talkative and “use fancy words” to “get some actual help from psychology.” The patient’s request to clinicians was that they should learn “how Sami express things,” described as being silent and not talking (Dagsvold et al., 2015). In the present study, a therapist stated that Sami patients adapt to the culture and context of mental health care, accepting verbal communication as a major part of therapy.

One could question to what extent mental health care can be adapted in accordance to patients’ cultures, and if it is desirable to provide mental therapy in accordance with the assumed Sami norm of not talking about emotions and mental problems. The results in this study indicate that clinicians experience Sami patients to be just as talkative as other patients.

Knowledge about Sami culture and societies in general serves as contextual knowledge but not as a truth explaining individual patients’ behavior and needs. The challenge for clinicians is to reflect on cultural issues and avoid taking general assessments about culture as a priori truths in therapy with Sami patients. Clinicians cannot assume that individual patients act in accordance with their assumptions about patients’ culture. Clinicians should keep an open mind and consider culture as a significant parameter in understanding the patient and the therapeutic interaction, including the impact of themselves in therapy with patients (American Psychological Association, 1990).

A large body of literature recommends that health professionals critically assess the implications of their theory of culture and how their understanding of culture may influence the mental health care they provide (Guarnaccia & Rodriguez, 1996; Kleinman & Benson, 2006; Vandenberg, 2010, p. 243). Most clinicians in our study, however, had not reflected on their theory of culture, nor on the discrepancies between their assumptions about Sami culture in general and their clinical experiences in the therapy room. The few clinicians who referred to clinical assessments of patients’ “cultural experiences” referred to the phenomenon of talking to the deceased. The clinicians emphasized the importance of exploring and clinically assessing the patient’s experiences. Clinical assessments of the possible delusional nature of talking with the deceased imply a decision as to whether it is a “normal cultural expression” or abnormal and thus a psychiatric symptom (Guarnaccia & Rodriguez, 1996). An essentialized idea of an ethnic group sharing cultural meanings may lead to mental health issues being mistaken for cultural differences (Vandenberg, 2010).

The clinicians in our study referred to the assessment process as balancing between different explanatory models. Kleinman and Benson (2006) suggest that health professionals should critically reflect on the impact of the explanatory model they use when making clinical assessments of patients’ mental health. The authors urge clinicians to perform critical self-reflection and examine their own position of “being between social worlds,” that is, the world of the clinician, the world of biomedicine, and the world of the patient. To achieve this, clinicians should be trained to perform cultural self-reflection and to consider the effects, not only of the culture of the patient but also of the culture of biomedicine that may delimit the assessment of patients’ experiences, recasting it into biomedical categories, without acquiring an understanding of the meaning of illness as experienced by the patient (Kleinman and Benson, 2006, p. 1675). The clinicians in our study did not report about exploring the experience of talking to the dead for its own sake, for example by determining who they are talking to, about what, and in what language. Here, assessing whether the Sami literally “talk” when they refer to talking with the dead might also be useful; it could, for example, mean having a sense of closeness or good memories of a deceased grandmother, thinking “What would she have done in a similar situation?”, or having a conversation in one’s head.

The responsibility to take the culture of patients into account is not exclusive to health professionals but is also important at the level of health organizations and institutions (Kirmayer, 2012). The clinicians in our study stated that they are in a position of power to decide whether a patient’s expression and experience are pathological or not. However, they reported a lack of team discussions about how to integrate culture into clinical practice.

The lack of professional discussions left the responsibility for understanding culture and determining its impact on the patient and the clinical encounter to the individual clinician. The clinicians in our study expressed a strong desire to take a patient’s cultural background into account. However, they reported a lack of knowledge of guidelines and professional training as to how to explore, assess, and operationalize cultural factors in clinical practice.

### Limitations

A different or broader demographic sample and other methodological approaches might have resulted in different findings. The study sample was limited to therapists in the northern Sami area because other institutions did not agree to participate. Clinicians working, for example, in Lule or southern Sami areas might have other experiences due to demographic, linguistic, individual, and contextual differences. The study results may not be applicable to mental health services for the entire Sami population in Norway.

The study was conducted in Norwegian because the interviewer (the first author) did not speak Sami sufficiently well to conduct interviews in Sami. A Sami-speaking and bilingual interviewer might have increased the recruitment of Sami-speaking participants and could have explored and discussed cultural issues in more detail with them. For Sami-speaking participants, the use of Norwegian may have limited the ability to speak freely. A broader sample and interviews in both Sami and Norwegian might have accessed other stories about clinical experiences and identified a broader range of meaning units associated with the impact of Sami culture on mental health care.

The study does not discuss the impact of clinician characteristics on the results. We did not recruit a strategic sample of therapists based on ethnicity, education, profession, or clinical approach preferences. We invited all the clinicians at the clinics to participate as they represented the offer Sami patients receive. We have information about the participants’ ethnicity, education, occupation, and time of residence; however, ethical considerations concerning anonymity restrained us from combining and revealing this information. Moreover, we lack information on their clinical approach, and we have no information about the participants’ patients’ opinions about the therapy sessions or topics discussed in this study.

This is an exploratory study. Therefore, we have illuminated some challenges, but cannot conclude about factors that impact on the participants’ clinical practices.

## Conclusion

This study aimed to explore Sami and non-Sami clinicians’ assumptions about Sami culture and how they provide “culturally adapted” mental health services to Sami patients. The results indicate that the incorporation of culture into mental health care is a complex process. The incorporation process requires reflection on the underlying culture concept, and an assessment of how culture and explanatory models affect clinicians' understanding of their patients. Also, attention to contextual differences and to the individual's needs and preferences is vital when considering integration of culture into mental health care. Moreover, the results indicate that most clinicians’, both Sami and non-Sami, descriptions of Sami culture were narrow and that their descriptions of Sami culture were dominated by “cultural traits” typically considered “different” and implicitly compared with an unspoken norm of “usual” or “normal.” We found that, when assessing the “normality” of practices like “communication with the deceased,” clinicians balanced between understanding such experiences as cultural phenomena and viewing them as symptoms of mental illness. Clinicians acknowledged the power dynamics inherent in such clinical assessments. Clinical assessments involving cultural phenomena were rarely a part of professional discussions. Consequently, the incorporation of culture into clinical practice was the individual clinician’s responsibility and therefore likely uneven.

One might not expect Sami clinicians to describe Sami culture in essentialized ways and report limited reflections about the impact of culture on therapy. However, the clinicians’ assumptions about Sami culture may be influenced by the media and public debate, where stereotypical descriptions of Sami culture dominate (Gaski, 2008; Kvidal-Røvik & Olsen, 2016; Mathisen, 2001). Furthermore, limited or no team discussions will not increase clinicians' knowledge and reflections on the culture's impact on mental health care; rather, the opposite.

A discrepancy existed between the participants’ descriptions of Sami culture (“they do not talk about mental problems”) and their experiences from clinical encounters with Sami patients (who do indeed talk about it). The discrepancy between assumptions about culture on a group level and individual preferences reminds us that “people have a wide range of opinions about how they put consensual culture into action: some are right in tune with consensual culture; others deviate from the cultural norm” (Matsumoto, 2006, p. 43). Knowledge about Sami culture and history is vital within mental health care but must be used with caution in clinical encounters with patients to avoid stereotyping and disregard of the individual patients’ needs and preferences.

The study has clinical implications. Health organizations, institutions, and professionals should contribute to a critical theorization of culture and aim for professional approaches to the integration of culture into mental health care. Health institutions should develop structures and settings for professional discussions on how to assess cultural phenomena. Cultural considerations should not be limited to the culture of “the others” but should rather include critical cultural self-reflection by clinicians and reflection on health care services as a whole.

The findings in this study should be followed up with studies of Sami patients’ experiences of receiving mental health care, exploring whether they feel that clinicians understand them when they express “cultural experiences.” Future research should include observational and fieldwork, examining clinicians’ assessments in more detail in clinical encounters with Sami patients, including team discussions on how to understand patients’ “cultural expressions and experiences.” Additionally, the impact of clinician characteristics on cultural considerations in therapy, such as profession, clinical experience, ethnicity, and training in cultural considerations, should be further investigated. This study had a small number of Sami-speaking participants and should be followed up by bilingual and Sami-speaking researchers to explore the impact of therapy language when discussing cultural issues.

In the present study, the participants expressed a need for professional discussions and development to provide better mental health services to Sami patients. One tool for improving mental health care to Sami patients is the Cultural Formulation Interview (CFI) DSM-5, originally published by the American Psychiatric Association (2016). A few of the participants had heard about the CFI, but none of them had used it in their clinics. Moreover, the CFI had not been translated to Sami at the time of the interviews for this study. However, the Sámi Norwegian National Advisory Unit on Mental Health and Substance Use (SANKS) is currently developing a Sami version of the CFI and plans to publish this version in Spring 2020. The translation of the CFI-DSM-5 to northern Sami language and the use of such interview guides might contribute to a better adaptation of the treatment offered to Sami patients by the Norwegian health care system. We recommend that the use of a Sami version of the CFI is monitored by research. Moreover, research on clinical assessment of culture should include considerations of the culture of the clinician as well as that of the mental health services.

## Notes

1 Several national laws, notably the Hospital Trust Act, Health and Care services Act, Sámi Act, and the Patients’ Rights Act, confirm Sami patients’ right to receive equitable health services. The Patients’ Rights Act specifies the right of all patients, including Sami, to receive health services, particularly information, adapted to individual needs, including cultural and linguistic background.

2 The Sami Language Administrative District covers the municipalities in which Sami patients, according to the Sami Act, have an extended right to use the Sami language in encounters with public services.

3 IRB file number 2010/2238/REK nord.
